# Vaginal microbiota of pregnant women with *Ureaplasma urealyticum* and *Mycoplasma hominis* infections

**DOI:** 10.3389/fcimb.2024.1445300

**Published:** 2024-09-09

**Authors:** Kwan Young Oh, Sunghee Lee, Jaewan Park, Mi Hye Park, Ji Hun Jeong, Jung Bo Yang, Chul Kwon Lim, Joong Gyu Ha, Yun Seok Yang

**Affiliations:** ^1^ Department of Obstetrics and Gynecology, School of Medicine, Eulji University, Daejeon, Republic of Korea; ^2^ Research Laboratories, ILDONG Pharmaceutical Co. Ltd., Hwaseong, Republic of Korea; ^3^ Department of Obstetrics and Gynecology, School of Medicine, Ewha Woman’s University, Seoul, Republic of Korea; ^4^ Department of Laboratory Medicine, School of Medicine, Eulji University, Daejeon, Republic of Korea

**Keywords:** vaginal microbiota, *Mycoplasma hominis*, *Ureaplasma urealyticum*, pregnant Korean women, preterm birth

## Abstract

**Background:**

The association between preterm birth and *Mycoplasma* species such as *Mycoplasma hominis* and *Ureaplasma urealyticum* has been extensively investigated. In a clinical setting, conventional diagnostic methods for them involve culture methods for *Mycoplasma* spp. and *Ureaplasma* spp., along with PCR tests. However, the clinical utility of these tests remains controversial, highlighting the necessity for more robust and reliable methods for identifying and understanding *Mycoplasma* infections.

**Objective:**

This study aimed to assess the distribution of microbiota in pregnant women with *Mycoplasma hominis* and *Ureaplasma urealyticum* infection by the comparison of conventional diagnostic methods with vaginal microbial community analysis.

**Study Design:**

This prospective case–control study involved 228 Korean pregnant women and utilized vaginal microbial community analysis, *Ureaplasma*/*Mycoplasma* culture, and 12-multiplex PCR for sexually transmitted diseases. Cross-correlation analysis in SPSS 27 compared the results of two conventional methods with vaginal microbial community analysis. R software generated box plots depicting the relative abundance of microorganisms. Network analysis was conducted using Cytoscape.

**Results:**

Positive *Ureaplasma urealyticum* culture findings were observed in 60.2% of patients, with 76.4% positive for *Ureaplasma parvum* PCR and 13.2% positive for *Ureaplasma urealyticum* PCR. *Mycoplasma hominis* culture was positive only in two patients, while *Mycoplasma hominis* PCR was positive in eight women. Vaginal microbial community analysis identified significant differences in relative abundances of *Gardnerella species* type I and *Fannyhessea vaginae* between the *Ureaplasma urealyticum* PCR positive and negative groups. *Mycoplasma hominis* PCR positive patients exhibited significant differences in 11 bacterial species, including *Gardnerella species* I and *Fannyhessea vaginae*.

**Conclusion:**

This study suggests that STD-PCR may be more accurate than *Ureaplasma*/*Mycoplasma* culture for the diagnosis of *Mycoplasma hominis* and *Ureaplasma urealyticum* infection. Also, the presence of *Gardnerella species* I and *Fannyhessea vaginae* implies their potential influences on *Ureaplasma urealyticum* and *Mycoplasma hominis* infections based on results of vaginal microbial community analysis. Therefore, vaginal microbial community analysis may give the more information of their pathophysiology.

## 1 Introduction

Preterm birth, defined as delivery before 37 weeks of gestation, poses a significant global public health issue and is a leading cause of perinatal mortality that can result in long-term health problems for surviving infants (Christopher et al., 2013; Jung et al., 2024). Intra-amniotic fluid infection, also termed intrauterine infection or chorioamnionitis, is a major contributor to preterm birth (Oh et al., 2022; Jung et al., 2024). The process leading to preterm birth often involves ascending infection, wherein microorganisms from the lower genital tract ascend into the amniotic cavity, inducing inflammation and potentially triggering premature labor (Oh et al., 2022; Jung et al., 2024). Intra-amniotic fluid infections are particularly associated with infections in the vaginal environment and have been linked to various microorganisms, including bacteria and *Mycoplasma* species (Alinezhad et al., 2022; Kwak et al., 2014; Matasariu et al., 2022).

The association between preterm birth and *Mycoplasma* species, such as *Ureaplasma urealyticum* (UU) and *Mycoplasma hominis* (MH), has been extensively investigated (Matasariu et al., 2022; Murtha and Edwards, 2014; Suzuki et al., 2018; Miyoshi et al., 2022). These microorganisms have been identified as potential pathogens associated with adverse pregnancy outcomes, including preterm birth. In particular, Miyoshi et al. claimed that positivity was independent predictive factor for preterm birth in patients with symptomatic threatened preterm labor (Miyoshi et al., 2022).

However, clinical diagnosis of vaginal infections caused by *Mycoplasma* strains presents challenges due to the predominance of opportunistic infections and difficulty in distinguishing pathogenic strains from commensal strains (Jonduo et al., 2022; Leli et al., 2018). There is still not conclusive evidence whether these microorganisms should be considered pathogens, or commensal floras (Leli et al., 2018).

Therefore, despite the known association between *Mycoplasma* infections and preterm birth, a notable gap exists in our understanding of the pathophysiology of these infections, hindering the development of effective clinical strategies beyond the traditional diagnostic methods of intra-amniotic fluid analysis through amniocentesis.

In a clinical setting, conventional diagnostic methods for *Mycoplasma* infections involve culture methods for *Mycoplasma* spp. and *Ureaplasma* spp., along with PCR tests for sexually transmitted diseases (STD-PCR) to detect these microorganisms in vaginal discharge. However, the clinical utility of these diagnostic tests remains controversial, highlighting the necessity for more robust and reliable methods for identifying and understanding *Mycoplasma* infections in pregnant women.

Recently, with the development of molecular genetic technology, vaginal microbial infection has been explained by changes in the vaginal microbial ecosystem (Ravel et al., 2011; Lee et al., 2020; Oh et al., 2021). In particular, metagenomics development such as vaginal microbial community analysis (VMCA) has given more information for vaginal infections such as bacterial vaginosis (BV) and aerobic vaginitis (AV) (Lee et al., 2020; Oh et al., 2021). Therefore, even in vaginal infections, which is the cause of intrauterine infection, is explained by the dysbiosis state in which this balance is broken in the biofilm concentrated in vaginal lactobacilli (Ravel et al., 2011; Lee et al., 2020; Oh et al., 2021). However, VMCA for MH and UU infections which are mentioned as independent factors of intrauterine infection, has not yet been reported. The results may be given the information for cofactors between pathologic infection and commercial flora of Mycoplasmas infections.

This study aims to contribute to existing knowledge by exploring the correlation between the vaginal microbiome, and *Mycoplasma* species infection. The comparison of conventional diagnostic methods with VMCA provides a comprehensive approach to understanding the distribution of microbiota in pregnant women with *Mycoplasma* infection, ultimately addressing the clinical implications of these findings.

## 2 Material and methods

### 2.1 Ethics approval

This study was approved by the Institutional Review Board (IRB) of Eulji University Hospital (IRB No. 2017-07-007-002 and 2020-01-011-002). All participants provided written informedconsent.

### 2.2 Study design and participants

A prospective case–control study was conducted on 228 pregnant Korean women, the same subjects as in a previous study (Lee et al., 2020). As described in our previous study, 16 women were excluded from analysis due to obstetric or medical illness (preeclampsia, five cases; gestational diabetes, seven cases; other medical diseases, four cases). In total, 212 women that fulfilled inclusion and exclusion criteria were selected.

Vaginal samples were obtained for VMCA, Gram staining for Nugent scoring, wet mounting for aerobic vaginitis (AV) score, *Ureaplasma*/*Mycoplasma* culture, and 12 multiplex STD-PCR test. However, the wet mount for diagnosis of AV was performed in 159 subjects of them because of the diagnostic limitation of requiring a fresh sample, and 12 multiplex STD-PCR testing was performed in 176 women because of sample condition.

### 2.3 Vaginal sampling

Sampling methods for VMCA, Gram staining, and wet mounting were performed, as previously described (Oh et al., 2021; Lee et al., 2020). Samples from the enrolled subjects were collected by a physician using a cotton swab for Gram staining and E-swab (Copan, Italia) for VMCA, from the posterior fornix of the vaginal wall. Samples for STD-PCR and *Ureaplasma/Mycoplasma* culture were obtained from the posterior fornix of the vaginal wall. VMCA samples were stored at −80°C until use. Samples for *Ureaplasma/Mycoplasma* culture and STD-PCR were transferred to a Green Cross laboratory.

### 2.4 *Ureaplasma/Mycoplasma* culture for UU and MH

**Figure 1:**
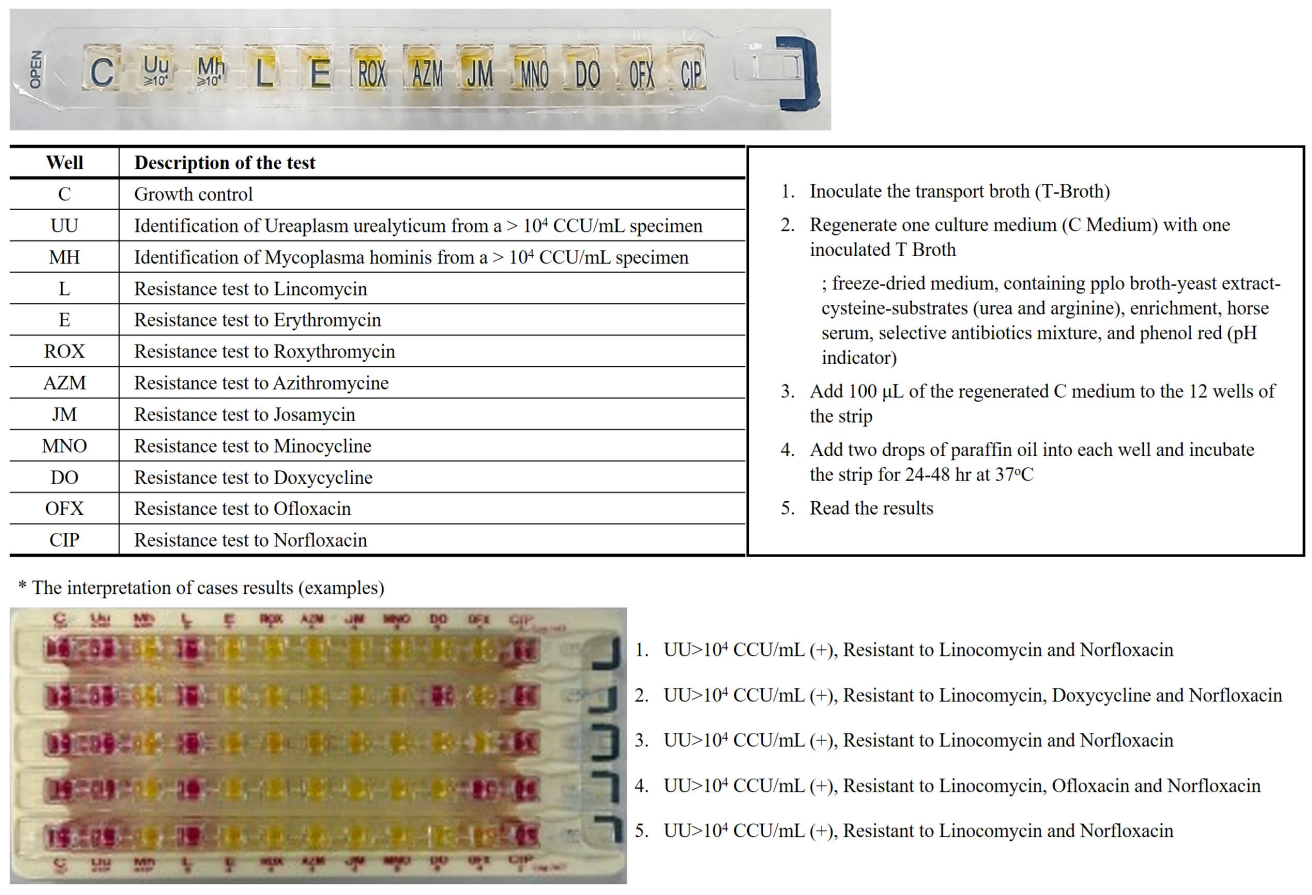
*Mycoplasma hominis/Ureaplasm urealyticum* culture method. On a single gallery, the identification, differential titration of UU and MH, testing for resistance to 9 antibiotics and growth control. The results are returned within 24 hours to 48 hours according to a colorimetric change in the wells going from orange-yellow to red/pink. (acid; (-) yellow color, alkali; (+) red color).

Vaginal swabs were obtained to detect MH and UU using the Mycoview Quantum^®^ kit (Zeakon Diagnostics, Besancon, France) following the manufacturer’s instructions (**Figure 1**). The test relies on specific metabolic properties and natural resistance of each *Mycoplasma* species: hydrolysis of urea and lincomycin resistance in UU and hydrolysis of arginine and erythromycin resistance in MH. Growth of these species is indicated by a color change of the pH indicator (Phenol red) from yellow (negative, acid) to red or pink (positive, alkali). The sample is incubated at 36 ± 1°C for 24 h, and the results are read. Positive results for UU were indicated by well 2, with significant levels ≥10^4^ CCU/mL, while MH was detected in well 3, with significant levels ≥10^4^ CCU/mL. Incubation can be continued for up to 48 h for MH identification; the color change is typically red and rarely appears as bright pink. The presence or absence of a red color indicated resistance or susceptibility to nine antimicrobial agents, respectively, according to the guidelines of the Clinical & Laboratory Standards Institute.

### 2.5 DNA extraction and 12 type STD-PCR

DNA extraction was performed, as previously described (Lee et al., 2020), and quantified using a NanoDrop spectrophotometer (NanoDrop Technologies, Inc., Wilmington, DE, USA). The extracted DNA samples underwent analysis for 12 STD pathogens (*Ureaplasma urealyticum* (UU)*, Ureaplasma parvum* (UP)*, Mycoplasma hominis* (MH), *Mycoplasma genitalium, Gardnerella species*(GS) previously known as *Gardnerella vaginalis* (Shvartsman et al., 2023), *Chlamydia trachomatis, Neisseria gonorrhoeae, Trichomonas vaginalis, Treponema pallidum, Candida albicans, Herpes simplex type I and type II*) detection with the GCdia™ STI-12 Fast Real-time PCR kit (Genes Laboratories, South Korea).

The 15 μl master mix was prepared by mixing 10 μl of 2x reaction mixture and 5 μl of the set primer/probe mix. Each set primer/probe mix detected four types of STD-related pathogens and provided an internal control. The extracted DNA sample (5 μl) was added to each master mix tube. PCR was conducted using the Bio-Rad CFX96 Real-Time PCR Detection System (Bio-Rad, Hercules, California (CA), USA), following conditions per manual guidelines.

### 2.6 Vaginal microbial community analysis

Metagenomics analysis was performed, as previously described (Lee et al., 2020). Briefly, DNA samples were processed using the QIAamp DNA Mini Kit (Qiagen, Hilden, Germany). The extracted DNA was quantified and PCR-amplified targeting the V3–V4 region of the 16S rRNA gene using Illumina adaptors. Barcoding was achieved through secondary amplification, confirmed by gel electrophoresis, and purified. DNA quality was assessed using a Bioanalyzer 2100 (Agilent Technologies Inc., Santa Clara, CA, USA). Sequencing was performed on an Illumina MiSeq platform (Illumina, San Diego, CA, USA) at ChunLab, Inc. De-multiplexed reads were analyzed using QIIME 2 and trimmed, merged, and filtered for low-quality reads based on a median quality score of Q25, and ambiguous base calls, as well as all chimeric sequences, were removed using the Deblur workflow (Amir et al., 2017; Bolyen et al., 2019). Multiple alignments and phylogenetic tree generation were performed using MAFFT and FastTree (Price et al., 2010; Katoh and Standley, 2013). Taxonomic analysis (80% identity) used the EzBioCloud database and BLAST+ consensus taxonomy classifier (Camacho et al., 2009).

### 2.7 Network analysis

Network analysis was conducted within the VMCA and PCR species using Cytoscape (version 3.10.1) (Shannon et al., 2003). Correlation significance, vaginal microbial community abundances, and three types of STD-PCR values were inputted and analyzed using an Edge-weighted Spring-embedded Layout. VMCA associations with STDs were represented as lines, with red indicating positive and blue indicating negative relationships. Line thickness represented the relative abundance (RA) of VMCA, whereas distance between nodes represented *p*-values of the PCR groups.

### 2.8 Statistical analysis

Statistical analyses were performed using SPSS Statistics ver. 27 (SPSS, Chicago, IL, USA). Correlation was analyzed using χ2 test and Fisher’s exact test, with significance set at *p* < 0.05. Demographic factors were compared between UU/MH culture-negative and -positive groups using the Mann–Whitney U-test (*p* < 0.05). In addition, 12 STD-PCR results were compared between groups.

We compared the *Ureaplasma/Mycoplasma* culture and STD-PCR results with those of VMCA using cross-correlation analysis in SPSS 27.0 (IBM, Chicago, IL, USA). The RA of VMCA for operative taxonomic units (OTUs) significantly differed between negative and positive UU, *Ureaplasma parvum* (UP), and MH PCR groups (*p* < 0.05) and was depicted as box plots using the ggplot2 package in R software (version 4.3.1) (Gustavsson et al., 2022).

## 3 Results

### 3.1 Correlation with *Ureaplasma/Mycoplasma* culture and STD-PCR results

In the 12 types of STD-PCR results, cases positive for *Mycoplasma genitalium* PCR, *Chlamydia trachomatis* PCR, and *Herpes Simplex virus (HSV)* type II were one each, and were thus excluded from analysis. **Table 1A** demonstrates that 60.2% (106/176) showed positive findings in *UU* culture. Of these, 81 patients (76.4%) tested positive for UP PCR and 14 patients (13.2%) tested positive for UU PCR (**Table 1A**). Additionally, 8 patients (7.5%) showed positive findings for MH PCR (**Table 1B**, **Supplementary Table 1B**). Cross-analysis between PCR results and culture test for all three strains showed statistically significant differences (*p* < 0.05). In addition, 84 patients (79.2%) tested positive for *Gardnerella species* (GS) PCR, and the *UU* culture-positive group showed significantly higher findings (*p* = 0.022). *Candida albicans* (CA) PCR also showed positive findings in 14 patients (13.2%), but unlike other PCR results, it did not show statistically significant differences between *UU* culture-positive and negative groups (**Table 1A**, **Supplementary Table 1A**).

**Table 1:** The dermographic characteristics and the results of sexually transmitted diseases (STD) PCR in *Ureaplasma urealyticum* (A)/*Mycoplasma hominis* (B) culture positive and negative groups.

(A)			
*Ureaplasma urealyticum*	Positive (N=106)	Negative (N=70)	*p*-value
Gravida	2.62 ± 3.25	2.54 ± 1.16	
Parity	1.01 ± 0.842	1.30 ± 0.89	
Maternal age (year, mean ± SD))	32.59 ± 4.32	33.63 ± 3.95	
Gestational age at delivery (week, mean ± SD))	38.05 ± 3.50	38.81 ± 1.81	
BV incidence	10(9.4%)	5(7.1%)	0.809
AV incidence	11(14.6%)	7(13.8%)	0.494
*Ureaplasma parvum* PCR (+) (n=83)	81 76.4%)	2(2.9%)	0.000*
*Ureaplasma urealyticum* PCR (+) (n=14)	14(13.2%)	0	0.001*
*Mycoplasma hominis* PCR (+) (n=8)	8(7.5%)	0	0.016*
*Mycoplasma genitalium* (+) (n=1)	0	1	0.398
*Gardnerella species* PCR (+) (n=129)	84(79.2%)	45(64.3%)	0.022*
*Candida albicans* PCR (+) (n=20)	14(13.2%)	6(8.6%)	0.243

(B)			
*Mycoplasma hominis*	Positive (N=2)	Negative (N=174)	*p*-value
Gravida	3.50 ± 0.707	2.58 ± 2.62	0.640
Parity	1.50 ± 0.707	1.12 ± 0.87	0.691
Maternal age (year, mean ± SD))	34.50 ± 3.54	32.99 ± 4.21	0.640
Gestational age at delivery (week, mean ± SD))	38.45 ± 1.20	38.36 ± 2.97	0.639
BV incidence	0	15(8.6%)	0.170
AV incidence	0	18(14.5%)	0.917
*Ureaplasma urealyticum* culture	2	104	0.361
*Ureaplasma parvum* PCR (+) (n=83)	2(100%)	81(46.6%)	0.221
*Ureaplasma urealyticum* PCR (+) (n=14)	0	14(8.0%)	0.847
*Mycoplasma hominis* PCR (+) (n=8)	2(100%)	6(3.4%)	0.002*
*Gardnerella vaginalis* PCR (+) (n=129)	2(100%)	127(73.0%)	0.536
*Candida albicans* PCR (+) (n=20)	1(50%)	19(10.9%)	0.215

BV, bacterial vaginosis; AV, aerobic vaginitis; * statistical significance (p < 0.05).

**Table 1B** and **Supplementary Table 1B** presents the results of comparative analysis between the MH STD-PCR results and culture-positive group. Among two MH PCR-positive cases, both showed positive results for UP and GS PCR. However, UP PCR (+) and GS PCR (+) showed no statistically significant differences between the MH culture groups. CA PCR (+) yielded positive findings in one patient (50%), and UU PCR was all negative, with no statistically significant difference between MH culture groups (**Table 1B**, **Supplementary Table 1B**).

Sensitivity and specificity of the UU/MH culture test with delayed STD-PCR results were calculated. The UU culture test showed sensitivity of 13.2% (14/106), specificity of 100% (70/70), predictive positive value of 100% (14/14), and predictive negative number of 43.2% (70/162). The MH culture showed sensitivity of 100% (2/2), specificity of 96.6% (168/174), predictive positive value of 25% (2/8), and predictive negative value of 96.6% (168/174). The UU culture test could not distinguish between UP and UU; therefore, the test was found to be specific but not sensitive, while the *MH* culture test had high sensitivity and specificity, but had a low predictive positive value at 25%.

### 3.2 Clinical characteristics of the STD-PCR positive group and correlation analysis

STD-PCR tests were performed to compare demographic and clinical characteristics between the positive (+) and negative (-) groups for UU, UP, MU, GV, and CA PCR, and correlations with other strains were analyzed. **Supplementary Table 1A** presents the clinical characteristics of the UU PCR (+) compared with the negative groups and PCR results of other strains. There were 14 UU PCR (+) patients, with no statistically significant difference in demographic and clinical characteristics when compared with the UU PCR (-) group of 162 patients.

Comparison of PCR results with other strains revealed only one UP PCR (+) patient in the UU PCR (+) group, with a statistically significant difference from the UU PCR (-) group (*p* = 0.001) (**Supplementary Table 1A**). MH PCR (+) in 1 patient (7.1%) did not show a statistically significant difference between UU PCR (+) and (-) groups. Also, in UU PCR (+) group, 13 GS (+) patients (92.9%) was confirmed, whereas 71.6% (116/160) of the UU (-) group also showed GV PCR (+) findings, with no statistically significant difference (*p* = 0.071). Two CA PCR (+) patients were observed in the UU PCR (+) group, with positive findings observed in 11.2% of the (-) group, indicating no correlation between the two groups.

**Table 1B** and **Supplementary Table 1B** presents the results for the MH PCR (+) group. The MH PCR (+) group included 8/176 patients (4.5%). Comparative analysis with the (-) group showed significantly higher incidences of bacterial vaginosis (BV) and AV (*p* = 0.000 and *p* = 0.006, respectively). All 8 patients in the MH PCR (+) group tested positive for GS PCR, but there was no statistical significance when compared with the negative group (*p* = 0.076). The frequency of UP PCR (+) was significantly higher than in the MH PCR (+) group than in the (-) group, but frequencies of GS PCR (+) and CA PCR (+) showed no significant difference. **Table 1A** and **Supplementary Table 1C** shows UP PCR results, showing the highest positivity rate at 47.2% (83/176). UU PCR (+) frequency was significantly lower in the UU PCR (+) group than in the negative group, whereas MH PCR (+) was significantly higher than in the negative group. Other strains did not exhibit any significant differences. CA PCR (+) was 11.3% (20/176). BV frequency was significantly higher in the CA PCR (+) group (*p* = 0.007), while AV frequency was not statistically significant. GS PCR (+) frequency was statistically significant at 95.0% (19/20) (*p* = 0.017). GS PCR(+) was 73.3% (129/176). In the GS PCR (+) group, BV (+) frequency was significantly higher at 10.9%, and CA PCR (+) frequency was also statistically significant at 11.4% (19/129).

### 3.3 Comparison of STD-PCR and VMCA results

**Figure 2:**
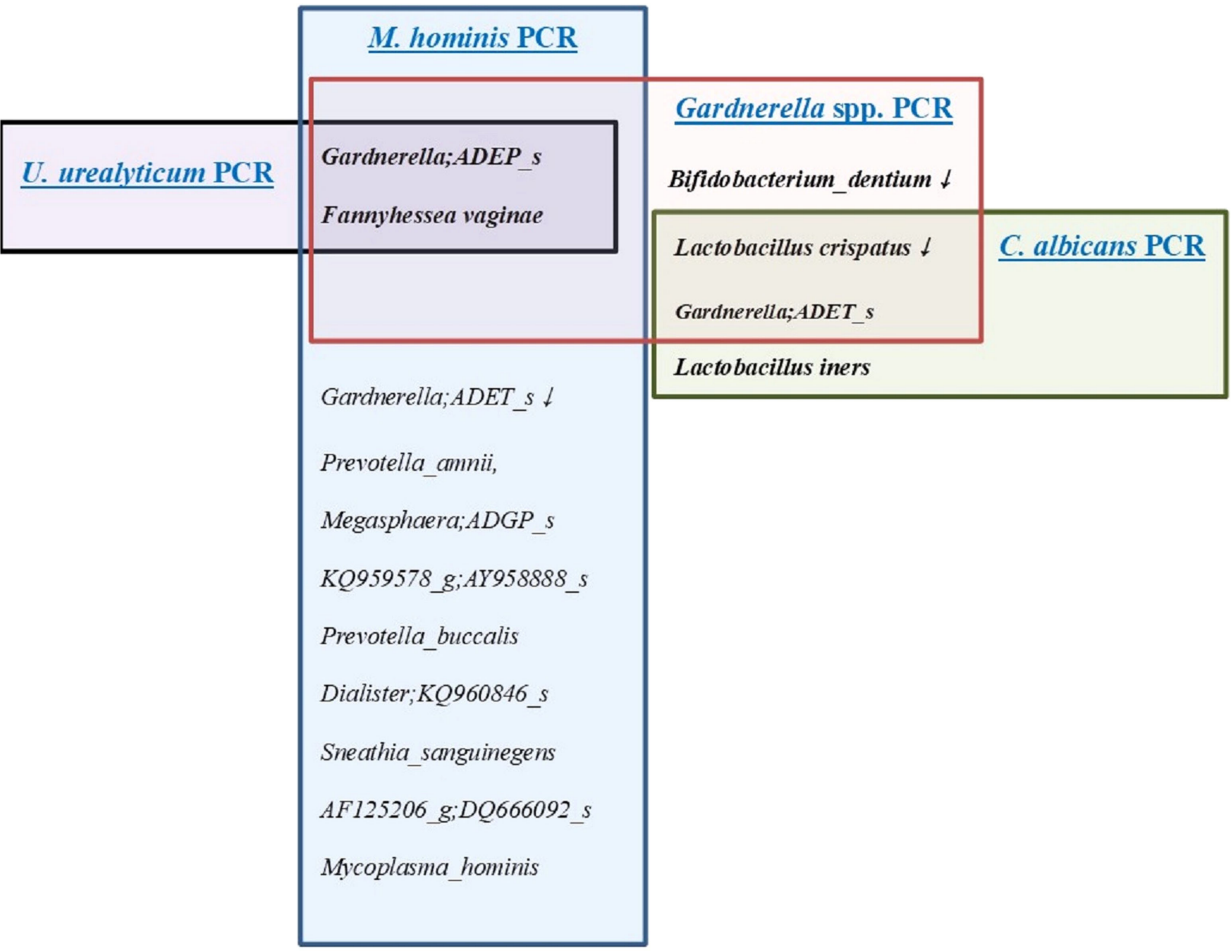
The distribution of microorganisms with a relative abundance of ≥1% in the results of the vaginal microbiome community analysis (VMCA) in women with positive results for four sexually transmitted diseases (STD) PCR (+) including *Ureaplasma urealyticum* (UU), *Mycoplasma hominis* (MH), *Gardnerella species* (GS), and *Candida albicans* (CA). - Black color line; microorganisms with a relative abundance of ≥1% in the results of the VMCA in women with positive result for UU PCR. - Blue color line; microorganisms with a relative abundance of ≥1% in the results of the VMCA in women with positive result for MH PCR. - Red color line; microorganisms with a relative abundance of ≥1% in the results of the VMCA in women with positive result for GS PCR. - Green color line; microorganisms with a relative abundance of ≥1% in the results of the VMCA in women with positive result for CA PCR.

VMCA comparison between UU PCR-positive and -negative patients revealed significant differences in GS type I (GS I*, Gardnerella; ADEP_s*) and *Fannyhessea vaginae* (FV), previously known as *Atopobium vaginae* (Nouioui et al., 2018) among species with RA of ≥1% (**Supplementary Table 2A**, **Figures 2**). Comparing the results of VMCA between the UU PCR-positive and -negative groups, OTUs with statistically significant differences in RA of 0.001% or more included 10 strains (**Supplementary Table 2A**), and GS I and FV showed significant differences in RA of 1% or higher (**Figures 2**). All these strains play important roles in BV. However, there was no significant difference in the diagnosis of BV between the two groups (**Supplementary Table 1A**). RA for UU showed significant difference between the UU PCR (-) and (+) groups, but remained less than 1%. UP showed significant differences between the two groups.

**Figure 3:**
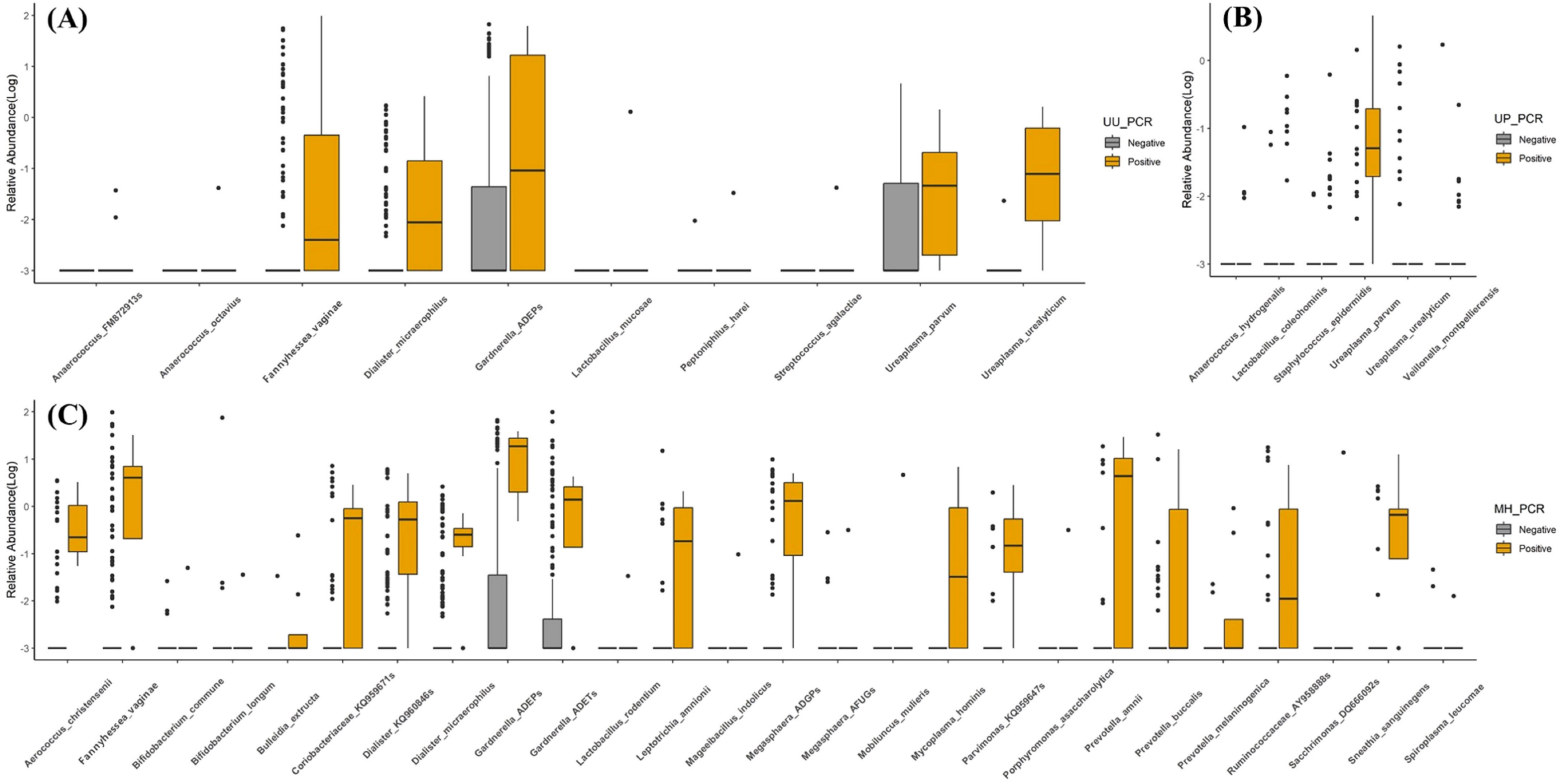
Observed vaginal microbiome community analysis (VMCA) species from negative or positive PCR detection group. **(A)** *Ureaplasma urealyticum* PCR, **(B)** *Ureaplasma parvum* PCR, and **(C)** *Mycoplasma hominis* PCR. All VMCA species showed significant differences between PCR-negative and -positive groups (*p* < 0.05).

**Figure 4:**
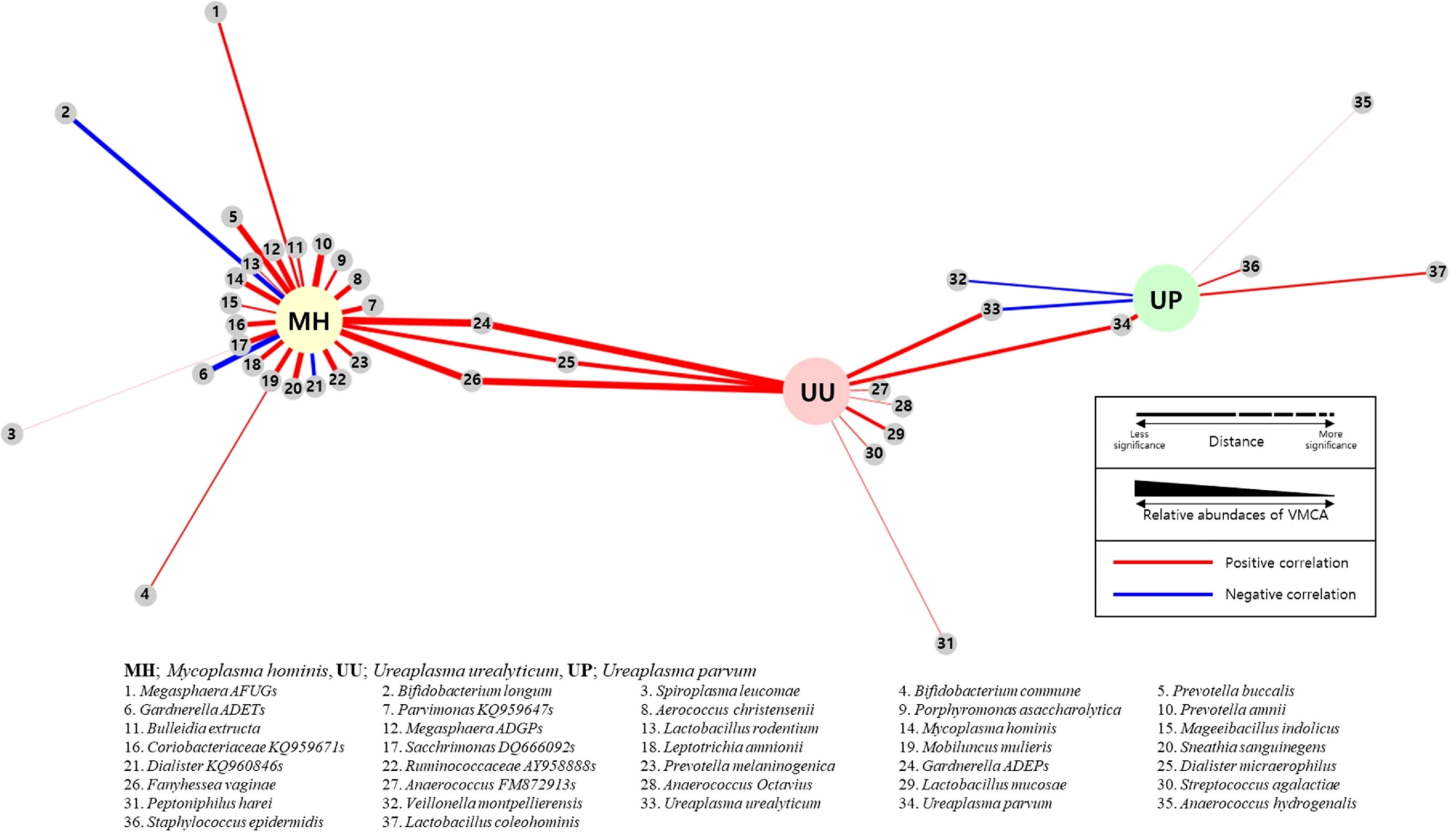
Network analysis between sexually transmitted diseases (STD) PCR and vaginal microbiome community analysis (VMCA) species. Each small gray node represents a VMCA species positively (red line) or negatively (blue line) correlated with MH, UU, and UP PCR with a *p*-value of <0.05. The closer the distance between VMCA and PCR species, the greater the statistical significance. The thickness of the line connecting VCMA and PCR species represents the relative abundance of VMCA. (MM, *Mycoplasma hominis*; UU, *Ureaplasma urealyticum*; UP, *Ureaplasma parvum)*.

Eight patients with MH PCR (+) showed significant differences in 26 VMCA species (RA> 0.001%) (**Supplementary Table 2B**, **Figures 3**). The species with RA of more than 1% included GS I, FV, *Gardnerella ADET_s(*GS II*)*, *Prevotella amnii*, *Megasphaera ADGP_s*, *AY95888_s*, *Prevotella buccalis*, *Dialister KQ960846_s*, *Sneathia sanguinegens*, *Sacchrimonas* DQ666092, and MH (**Figures 2**–**4**). GS I, FA, and GS II among them were known as strains associated with BV.

There was no statistically significant strain between the UP PCR (+) and (-) groups at an RA of ≥1%, whereas six strains showed statistically significant differences at RA of ≥0.001% (**Supplementary Table 2B**). The RA of UP was significantly higher in the UP (+) group, while that of UU was significantly lower. Four strains were significantly elevated in the UP (+) group.

In the GS PCR (+) group, 26 OTUs (0.001%) showed statistically significant differences when compared with the (-) group, with 5 OTUs having an RA of ≥1% (**Supplementary Table 2D**, **Figure 2**). *Lactobacillus crispatus* and *Bifidobacterium dentium* were significantly higher in the GS PCR (-) group (**Figure 2**). Conversely, the other three OTUs (GS I, GS II, and FV) were significantly higher in the GS (+) group, while *Lactobacillus iners* showed no significant difference (**Figure 2**).

Seven strains with an RA of > 0.001% in VMCA results showed significant differences between the CA PCR (+) and (-) groups, with *L. crispatus, L. iners*, and GS II showing RA > 1%. *L. crispatus* was more prevalent in the (-) group, while *L. iners* and GS II were more common in the CA PCR (+) group (**Supplementary Table 2E**).

**Figure 2** shows OTUs with an RA of >1% in each strain PCR (+) group. In the three-strain PCR (UU PCR, MH PCR, and GS PCR), the strains that emerged significantly in the (+) group were GS I and FV. Moreover, in GS PCR and CA PCR (+), *L. crispatus* decreased significantly, while GS II increased significantly. For evaluation of relationship among these OTUs, network analysis was done (**Figure 4**). By it, we could know that UU and MH PCR (+) were strongly related with higher RA of GS 1, 2 and FV of VMCA.

## 4 Discussion

Vaginal microbiota is associated with women’s reproductive health, particularly during pregnancy (Ravel et al., 2011; Romero et al., 2014; Walther-Antonio et al., 2014; Lee et al., 2020). During pregnancy, the vaginal microbiota shows a further strengthening of the *Lactobacillus* spp. dominant condition (Walther-António et al., 2014). It has been reported that when this *Lactobacillus* dominant condition is destroyed, vaginal infection occurs and leads to adverse perinatal outcomes (Hay et al., 1994; Goldenberg et al., 2008; Koumans et al., 2007; Goldenberg et al., 2008). Vaginitis reported to be related to premature birth includes BV, AV, and Mycoplasma infection. The association between preterm birth and Mycoplasma species, such as UU and MH has been extensively investigated (Alinezhad et al., 2022; Jonduo et al., 2022; Kwak et al., 2014; Matasariu et al., 2022; Miyoshi et al., 2022). In particular, UU (+) in amniotic fluid was significantly more detected in women with preterm labor (Yoon et al., 1998; DiGiulio, 2012). Moreover, it reported as an independent factor in the occurrence of preterm birth (Miyoshi et al., 2022). Despite the known association between Mycoplasma infections and preterm birth, a notable gap exists in our understanding of the pathophysiology of these infections, hindering the development of effective clinical strategies beyond the traditional diagnostic methods of intra-amniotic fluid analysis through amniocentesis. Therefore, we tried to analyze the distribution of microbiota in the vagina using new technology with the conventional diagnostic method such as culture and STD-PCR to examine the pathophysiology of these strains.

This study yielded several key findings. First, *Ureaplasma*/*Mycoplasma* culture showed a very high prevalence of UU (+) at 60.2% (106/176). As a result of comparing these results with STD-PCR results, UP PCR positivity was 76.4% and UU PCR positivity was 13.2%. This suggests limitations of UU culture in distinguishing between UP and UU, as UP was also diagnosed as UU. MH culture (+) prevalence was very low at 1.1%, with only 2 patients showing positive MH culture out of 8 positive MH PCR cases. Despite the high diagnostic specificity of 100%, the diagnostic value was not high, with a predictive positive value of 25% (2/8). Evaluation based on STD-PCR results revealed low sensitivity (14/106, 13.2%) and predictive negative value (70/162, 43.2%) for UU culture, but specificity (100%, 70/70) and predictive positive values (100%, 14/14) were very high. The sensitivity of the MH culture test was found to be very high (100%, 2/2), but predictive positive values (25%, 2/8) were low.

Detection and biovar discrimination of UU was reported by several researchers (Gupta et al., 2008; Robertson and Stemke, 1982) after biomolecular genetic technologies such as PCR. UP has been reported as more likely to be a commensal strain than UU in several studies (Gupta et al., 2008; Bao et al., 2010; Humburg et al., 2012), suggesting a potential overdiagnosis of UU infection using the *Ureaplasma*/*Mycoplasma* kit. Conversely, diagnosis by MH culture kit shows high sensitivity, but the predictive positive value is very low at 25%, suggesting the possibility of missed diagnosis. By those we knew that culture method had these like disadvantages for diagnosis of UU/MH infection although it gave very useful information on sensitive antibiotics. Therefore, *Ureaplasma*/*Mycoplasma* culture may not be appropriate as primary diagnostic method, while could serve as a secondary diagnostic or supportive method to STD-PCR, providing valuable information on sensitive antibiotics and enhancing diagnosis.

In the 12 types of STD-PCR tests, UU PCR prevalence was 8.0%, UP PCR prevalence was 47.2%, MH PCR prevalence was 4.5%, GV prevalence was 73.3%, and CA prevalence was 11.4% (**Supplementary Table 1**). As a result of conducting VMCA between STD-PCR positive and negative groups, strains with an RA of >1% that were notably present in three strain PCRs (UU PCR, MH PCR, and GS PCR) positive groups were GS I and FV (**Figure 2**). In both GS PCR (+) and CA PCR (+) groups, *L. crispatus* decreased significantly and GS II increased significantly (**Figure 2**).

As reported in many researches, it is known that *Lactobacillus spp*, especially *L. crispatus*, is dominant in vaginal discharge in healthy pregnant women, while in the discharge of women with vaginal dysbiosis such as BV and AV, various strains are mixed without dominant *L. species* (Ravel et al., 2011; Lee et al., 2020; Oh et al., 2021). Additionally, many researchers have reported that GS plays a role in helping women’s vaginal microbial infections (Morrill et al., 2020; Randis and Ratner, 2019; Morrill et al., 2020). Our results are similar as those of other researches.

The high prevalence of UP PCR (+) and GS PCR (+) may require the distinction between pathogenic and commensal strains. GS is classified into several genotypes, with diverse pathophysiologies (Ingianni et al., 1997; Castro et al., 2020; 2020; Vaneechoutte et al., 2019). In our study, two strains were confirmed as positive in several patients diagnosed with normal flora in Gram staining and wet smear tests. However, in UU PCR (+), MH PCR (+), and GS PCR (+) groups, VMCA showed significantly higher RA of GS I, while GS II showed high RA in GS PCR (+) and CA PCR (+) groups (**Figure 2**). Therefore, GS may plays a role in the development of vaginal infection. However, because GS was found to exist as a commensal strain, it is necessary to study its pathophysiology to identify conditions where it acts as a pathogenic strain. Therefore, when these strains are positive alone, it cannot be diagnosed as vaginitis.

UP has been accepted as a commensal strain in several studies (Ingianni et al., 1997). To confirm this, we compared and analyzed the RA of strains between the STD PCR (+) and (-) groups using VMCA. We found no strains with significant differences in RA of >1%, with only six strains showing differences in RA of >0.001%. The largest difference was observed in UP. Subsequently, UU, *Veillonella montpellierensis*, and the others followed (**Supplementary Table 2B**, **Figures 3**, **4**). This suggests that UP alone is unlikely to cause vaginal dysbiosis and does not provide evidence on whether it promotes the growth of other strains.

In the UU PCR (+) group, only GV I and FV showed differences at an RA of ≥1%, which were. In the MH PCR (+) group, 11 strains showed significant differences at an RA of ≥1%. Strains related to BV include GS I, FV, and GS II (**Figure 2**).

Several studies have reported the effects of MH on preterm birth (Alinezhad et al., 2022; Jonduo et al., 2022; Sprong et al., 2020), and it has also been reported that MH may be a component of BV (Abou Chacra et al., 2022; Cox et al., 2016). Considering the results of our study, it is difficult to believe that it is capable of independently causing vaginal dysbiosis, and it seems reasonable to view it as a strain related to BV. However, since the frequency in our study was low, further research is necessary.

In the GS PCR (+) group, 26 OTUs (0.001%) showed significant differences when compared with the (-) group, with 5 OTUs having an RA of ≥1% (**Supplementary Table 2D**, **Figure 2**). *L. crispatus* and *B. dentium* were also significantly higher in the GS PCR (-) group (**Figure 2**). However, the other three OTUs (GS I, GS II, FV) were significantly higher in the GS (+) group (**Figure 2**). As reported in several studies, GS is a representative strain of vaginal dysbiosis, such as BV and AV (Donders et al., 2002; Machado and Cerca, 2015; Morrill et al., 2020). Some studies have reported that GS plays an important role in allowing other pathological strains to pass through the cervical barrier (Gerson et al., 2022; Sierra et al., 2018), and has also been reported to reveal different characteristics depending on the genotype (Janulaitiene et al., 2017; Schellenberg et al., 2016; Castro et al., 2020; Vaneechoutte et al., 2019). We can’t see the correlation between GS (+) group and preterm birth, however, it may be related to antibiotic treatment administered to mothers diagnosed with BV/AV during early pregnancy in this study.

CA PCR showed a statistically significant difference between the (+) and (-) groups in seven strains with an RA of ≥0.001%, among which *L. crispatus*, *L. iners*, and GS II showed an RA of ≥1%. *L. crispatus* was more common in the (-) group, but *L. iners* and GS II were more prevalent in the CA PCR (+) group (**Figure 2**, **Supplementary Table 2E**).

Strains with an RA of ≥1% significantly emerged in all three strain PCR (UU PCR, MH PCR, and GS PCR) positive groups, were GS I and FV (**Figures 2**–**4**). In both GS PCR (+) and CA PCR (+) group, *L. crispatus* decreased significantly and GS II increased significantly (**Figure 2**). Through these results, we were able to know that GS I and FV strains play an important role in Mycoplasma infection. In addition, *L.crispatus* plays an important defensive role in GS and CA infection. GS, MH, and UU infections have been known to be important causes of premature birth in pregnant women. Also, several studies have reported an association between CA and preterm birth (Schuster et al., 2020; Maki et al., 2017; Payne et al., 2016). CA, like many other opportunistic bacteria, is reported to exist as a commensal bacteria, but it has been thought that it can forms a biofilm and act as a pathogen due to various factors (Wu et al., 2020; Takano et al., 2023). However, further research is needed. In our study, there was no significant difference in preterm birth between the CA PCR (+) and (-) groups as antifungal vaginal suppository treatment was used when CA was diagnosed by STD-PCR

MH and UU infections may affect the presence of GS I and FV. Therefore, *Mycoplasma* infection may have clinical significance on the distribution of vaginal microbiota. The underlying mechanism must be investigated in the future. Also, VMCA may be more informative diagnostic tool than conventional methods such as culture and PCR.

### 4.1 Strength and limitations

This study has very important clinical value as it is the first to analyze VMCA to understand the pathophysiology of MH and UU infection, which are important strains in premature birth. However, the limitation of this study is that the number of study subjects was too small to analyze the relationship between the preterm birth group and them. Therefore, further studies are needed on this topic.

## 5 Conclusion

The diagnosis of *Mycoplasma* infection is more accurate in STD-PCR than in *Ureaplasma/Mycoplasma* cultures. Additionally, new technologic method, VMCA may be more informative tool for understanding pathophysiology of MH and UU infection. By it we can know that the presence of GS I and FV implies their potential influences on MH and UU infection.

## Data Availability

The datasets generated in this study can be found in European Nucleotide Archive (ENA) using the accession number PRJEB34614 (https://www.ebi.ac.uk/ena/browser/view/PRJEB34614).
