# Supplementary material for: Vaginal microbiota of pregnant women with Ureaplasma urealyticum and Mycoplasma hominis infections
Source: Front Cell Infect Microbiol. 2024 Sep 9;14:1445300. doi: 10.3389/fcimb.2024.1445300 (PMC11417019; doi:10.3389/fcimb.2024.1445300)
Supplement: Supplementary file 1 [file Table1.docx]

**Supplementary Table 1. The PCR results of sexually transmitted disease (STD)-causing bacteria including: (A) *Ureaplasma urealyticum*, (B) *Mycoplasma hominis*, (C) *Ureaplasma parvum*, (D) *Candida albicans*, and (E) *Gardnerella vaginalis*.**

(A)

| ***Ureaplasma urealyticum*** | **Negative (N=162)** | **Positive (N=14)** | ***p*-value** |
| --- | --- | --- | --- |
| Gravida | 2.58+2.67 | 2.64+1.69 | 0.672 |
| Parity | 1.11+0.859 | 1.36+1.01 | 0.366 |
| Maternal age (year, mean +SD) | 32.85+4.187 | 34.79+4.042 | 0.097 |
| Gestational age at delivery (week, mean +SD) | 38.270+3.045 | 39.329+1.455 | 0.135 |
| BV incidence | 14(8.6%) | 1(7.1%) | 0.864 |
| AV incidence | 15(12.9%) | 3(30%) | 0.357 |
| *Mycoplasma hominis* culture (n=2) | 2 | 0 | - |
| *Ureaplasma urealyticum* culture (n=106) | 92 | 14 | - |
| *Ureaplasma parvum* PCR (+) (n=83) | 82(50.6%) | 1(7.1%) | 0.001* |
| *Mycoplasma hominis* PCR (+) (n=8) | 7(4.3%) | 1(7.1%) | - |
| *Mycoplasma genitalium* PCR (+) (n=1) | 1 | 0 | - |
| *Gardnerella species* PCR (+) (n=129) | 116(71.6%) | 13(92.9%) | 0.071 |
| *Candida albicans* PCR (+) (n=20) | 18(11.1%) | 2(14.3%) | 0.491 |

(B)

| ***Mycoplasma hominis*** | **Negative (N=168 )** | **Positive (N=8)** | ***p*-value** |
| --- | --- | --- | --- |
| Gravida | 2.60+2.65 | 2.38+1.41 | 0.965 |
| Parity | 1.11+0.87 | 1.38+0.917 | 0.895 |
| Maternal age (year, mean +SD) | 32.92+4.20 | 34.88+4.05 | 0.996 |
| Gestational age at delivery(week, mean +SD) | 38.42+2.71 | 37.15+6.34 | 0.238 |
| BV incidence | 10(6.0%) | 5(62.5%) | 0.000* |
| AV incidence moderate | 14(11.9%) | 4(50%) | 0.006* |
| *Mycoplasma hominis* culture (+) (n=2) | 0 | 2 | - |
| *Ureaplasma urealyticum* culture (+) (n=106) | 98 | 8 | - |
| *Ureaplasma parvum* PCR (+) (n=83) | 76(45.2%) | 7(87.5%) | 0.027* |
| *Ureaplasma urealyticum* PCR (+) (n=14) | 13(7.7%) | 1(12.5%) | 0.492 |
| *Gardnerella species* PCR (+) (n=129) | 121(72.0%) | 8(100%) | 0.111 |
| *Candida albicans* PCR (+) (n=20) | 18(10.7%) | 2(25.0%) | 0.226 |

(C)

| ***Ureaplasma parvum*** | **Negative (N= 93)** | **Positive (N=83 )** | ***p-*value** |
| --- | --- | --- | --- |
| Gravida | 2.80+3.39 | 2.35+1.15 | 0.256 |
| Parity | 1.24+0.914 | 1.00+0.81 | 0.074 |
| Maternal age (year, mean +SD) | 33.59+4.11 | 32.35+4.22 | 0.650 |
| Gestational age at delivery(week, mean +SD) | 38.645+2.73 | 38.02+3.08 | 0.173 |
| BV incidence | 8(8.6%) | 7(8.4%) | 0.754 |
| AV incidence moderate | 9(14.1%) | 9(14.5%) | 0.333 |
| *Mycoplasma hominis* culture (+) (n=2) | 0 | 2 | - |
| *Ureaplasma urealyticum* culture (+) (n=106) | 25 | 81 | - |
| *Ureaplasma urealyticum* PCR (+) (n=14) | 13(14.0%) | 1(1.2%) | 0.001 |
| *Mycoplasma hominis* PCR (+) (n=8) | 1(1.1%) | 7(8.4%) | 0.027* |
| *Gardnerella species* PCR (+) (n=129) | 65(69.9%) | 64(77.1%) | 0.280 |
| *Candida albicans* PCR (+) (n=20) | 11(11.8%) | 9(10.8%) | 0.837 |

(D)

| ***Candida albicans*** | | **Negative (N=156 )** | | **Positive (N= 20)** | | ***p*-value** | |
| --- | --- | --- | --- | --- | --- | --- | --- |
| Gravida | | 2.60+2.74 | | 2.50+1.05 | | 0.875 | |
| Parity | | 1.10+0.86 | | 1.30+0.92 | | 0.377 | |
| Maternal age (year, mean +SD) | | 32.88+4.21 | | 34.00+4.05 | | 0.257 | |
| Gestational age at delivery(week, mean +SD) | | 38.38+3.06 | | 38.18+1.95 | | 0.712 | |
| BV incidence (n=15) | | 13(8.3%) | | 2(10.0%) | | 0.007* | |
| AV incidence (> moderate) | | 16(14.6%) | | 2(11.8%) | | 0.789 | |
| *Mycoplasma hominis* culture (n=2) | | 1 | | 1 | | - | |
| *Ureaplasma urealyticum culture* (n=106) | | 92 | | 14 | | - | |
| *Ureaplasma parvum* PCR (+) (n=83) | | 74(47.4%) | | 9(45.0%) | | 0.837 | |
| *Ureaplasma urealyticum* PCR (+) (n=14) | | 12(7.7%) | | 2(10.0%) | | 0.663 | |
| *Mycoplasma hominis* PCR (+) (n=8) | | 6(3.8%) | | 2(10.0%) | | 0.226 | |
| *Mycoplasma genitalium* PCR (+)(n=1) | | 1(0.6%) | | 0 | | 0.720 | |
| *Gardnerella species* PCR (+) (n=129) | 110(70.5%) | | 19(95.0%) | | 0.017* | |  |

(E)

| ***Gardnerella vaginalis*** | **Negative (N=47 )** | **Positive (N= 129)** | ***p*-value** |
| --- | --- | --- | --- |
| Gravida | 2.53+1.18 | 2.61+2.97 | 0.868 |
| Parity | 1.23+0.96 | 1.09+0.84 | 0.323 |
| Maternal age (year, mean +SD) | 33.81+4.17 | 32.71+4.18 | 0.126 |
| Gestational age at delivery (week, mean +SD) | 38.04+3.73 | 38.48+2.62 | 0.474 |
| BV incidence | 1(2.1%) | 14(10.9%) | 0.012* |
| AV incidence | 1(3.7%) | 17(17.2%) | 0.186 |
| *Mycoplasma hominis* culture (n=2) | 0 | 2 | **-** |
| *Ureaplasma urealyticum* culture (n=106) | 22 | 84 | **-** |
| *Ureaplasma parvum* PCR (+) (n=83) | 19(40.4%) | 64(49.6%) | 0.309 |
| *Ureaplasma urealyticum* PCR (+) (n=14) | 1(2.1%) | 13(10.1%) | 0.117 |
| *Mycoplasma hominis* PCR (+) (n=8) | 0 | 8(6.2%) | 0.111 |
| *Candida albicans* PCR (+) (n=20) | 1(2.1%) | 19(11.4%) | 0.017* |

BV: bacterial vaginosis, AV: aerobic vaginitis, *** statistical significance (*p* < 0.05)

**Supplementary Table 2. Positive and negative detection of vaginal microbial community for sexually transmitted diseases-associated bacteria and fungi, including (A) *Ureaplasma urealyticum*, (B) *Ureaplasma parvum*, (C) *Mycoplasma hominis*, and (D) *Gardnerella vaginalis*,**

(A)

| ***Ureaplasma urealyticum*** | Negative (N=162) | Positive (N=14) | *p*-value |
| --- | --- | --- | --- |
| *Bacteria;Actinobacteria;Actinobacteria_c;Bifidobacteriales;*  *Bifidobacteriaceae;Gardnerella;ADEP_s* | 3.425±9.989 | 12.667±21.052 | 0.035 |
| *Bacteria;Actinobacteria;Coriobacteriia;Coriobacteriales;*[*Atopobiaceae*](https://en.wikipedia.org/wiki/Atopobiaceae)*; Fannyhessea ; Fannyhessea vaginae* | 1.797±7.349 | 10.862±28.272 | 0.025 |
| *Bacteria;Tenericutes;Mollicutes;Mycoplasmatales;Mycoplasmataceae_f1;*  *Ureaplasma;Ureaplasma_parvum* | 0.143±0.491 | 0.184±0.373 | 0.029 |
| *Bacteria;Firmicutes;Negativicutes;Veillonellales;Veillonellaceae;Dialister;*  *Dialister_micraerophilus* | 0.076±0.263 | 0.346±0.738 | 0.006 |
| *Bacteria;Tenericutes;Mollicutes;Mycoplasmatales;Mycoplasmataceae_f1;*  *Ureaplasma;Ureaplasma urealyticum* | 0.000±0.001 | 0.351±0.490 | 0.000 |
| *Bacteria;Firmicutes;Bacilli;Lactobacillales;Lactobacillaceae;Lactobacillus;*  *Lactobacillus_mucosae* | 0.000 | 0.092±0.345 | 0.001 |
| *Bacteria;Firmicutes;Bacilli;Lactobacillales;Streptococcaceae;Streptococcus;*  *Streptococcus_agalactiae* | 0.000 | 0.003±0.011 | 0.001 |
| *Bacteria;Firmicutes;Tissierellia;Tissierellales;Peptoniphilaceae;*  *Anaerococcus;FM872913_s* | 0.000 | 0.003±0.010 | 0.000 |
| *Bacteria;Firmicutes;Tissierellia;Tissierellales;Peptoniphilaceae;*  *Peptoniphilus;Peptoniphilus_harei* | 0.000 | 0.002±0.008 | 0.027 |
| *Bacteria;Firmicutes;Tissierellia;Tissierellales;Peptoniphilaceae;*  *Anaerococcus;Anaerococcus_octavius* | 0.000 | 0.002±0.011 | 0.001 |

(B)

| ***Ureaplasma parvum*** | Negative (N=932) | Positive (N=83) | *p*-value |
| --- | --- | --- | --- |
| *Bacteria;Tenericutes;Mollicutes;Mycoplasmatales;Mycoplasmataceae_f1;*  *Ureaplasma;Ureaplasma_parvum* | 0.027±0.155 | 0.280±0.659 | 0.000 |
| *Bacteria;Tenericutes;Mollicutes;Mycoplasmatales;Mycoplasmataceae_f1;*  *Ureaplasma;Ureaplasma_urealyticum* | 0.053±0.223 | 0.000±0.000 | 0.001 |
| *Bacteria;Firmicutes;Negativicutes;Veillonellales;Veillonellaceae;*  *Veillonella;Veillonella_montpellierensis* | 0.018±0.177 | 0.003±0.024 | 0.021 |
| *Bacteria;Firmicutes;Bacilli;Lactobacillales;Lactobacillaceae;*  *Lactobacillus;Lactobacillus_coleohominis* | 0.001±0.010 | 0.018±0.077 | 0.030 |
| *Bacteria;Firmicutes;Bacilli;Bacillales;Staphylococcaceae;Staphylococcus;Staphylococcus epidermidis* | 0.000±0.001 | 0.009±0.068 | 0.008 |
| *Bacteria;Firmicutes;Tissierellia;Tissierellales;Peptoniphilaceae;*  *Anaerococcus;Anaerococcus_hydrogenalis* | 0.000 | 0.001±0.011 | 0.033 |

(C)

| ***Mycoplasma hominis*** | Negative ( n=168 ) | Positive (n=8 ) | *p*-value |
| --- | --- | --- | --- |
| *Bacteria;Actinobacteria;Actinobacteria_c;Bifidobacteriales;*  *Bifidobacteriaceae;Gardnerella;ADEP_s* | 3.524±10.907 | 17.518±14.957 | 0.000 |
| *Bacteria;Actinobacteria;Coriobacteriia;*[*Atopobiaceae*](https://en.wikipedia.org/wiki/Atopobiaceae)*;Fannyhessea; Fannyhessea vaginae* | 2.220±10.599 | 8.776±12.272 | 0.000 |
| *Bacteria;Actinobacteria;Actinobacteria_c;Bifidobacteriales;*  *Bifidobacteriaceae;Gardnerella;ADET_s* | 1.825±9.631 | 1.635±1.491 | 0.001 |
| *Bacteria;Bacteroidetes;Bacteroidia;Bacteroidales;Prevotellaceae;Prevotella;Prevotella_amnii* | 0.248±1.768 | 7.711±10.106 | 0.000 |
| *Bacteria;Actinobacteria;Actinobacteria_c;Bifidobacteriales;*  *Bifidobacteriaceae;Bifidobacterium;Bifidobacterium_longum* | 0.449±5.820 | 0.004±0.012 | 0.046 |
| *Bacteria;Firmicutes;Negativicutes;Veillonellales;Veillonellaceae;Megasphaera;ADGP_s* | 0.337±1.323 | 1.882±1.870 | 0.000 |
| *Bacteria;Firmicutes;Clostridia;Clostridiales;*  *Ruminococcaceae;KQ959578_g;AY958888_s* | 0.319±2.078 | 1.425±2.700 | 0.000 |
| *Bacteria;Bacteroidetes;Bacteroidia;Bacteroidales;Prevotellaceae;Prevotella;Prevotella_buccalis* | 0.260±2.673 | 3.166±6.088 | 0.001 |
| *Bacteria;Fusobacteria;Fusobacteria_c;Fusobacteriales;*  *Leptotrichiaceae;Sneathia; Leptotrichia_amnionii* | 0.114±1.173 | 0.604±0.813 | 0.000 |
| *Bacteria;Firmicutes;Negativicutes;Veillonellales;Veillonellaceae;*  *Dialister;KQ960846_s* | 0.157±0.804 | 1.138±1.687 | 0.000 |
| *Bacteria;Actinobacteria;Coriobacteriia;Coriobacteriales;*  *Coriobacteriaceae;KQ959671_g;KQ959671_s* | 0.158±0.832 | 0.798±1.002 | 0.000 |
| *Bacteria;Fusobacteria;Fusobacteria_c;Fusobacteriales;*  *Leptotrichiaceae;Sneathia;Sneathia_sanguinegens* | 0.051±0.330 | 2.025±4.296 | 0.000 |
| *Bacteria;Firmicutes;Bacilli;Lactobacillales;Aerococcaceae;*  *Aerococcus;Aerococcus_christensenii* | 0.087±0.442 | 0.815±1.132 | 0.000 |
| *Bacteria;Firmicutes;Tissierellia;Tissierellales;Peptoniphilaceae;*  *Parvimonas;KQ959647_s* | 0.016±0.155 | 0.730±1.131 | 0.000 |
| *Bacteria;Firmicutes;Negativicutes;Veillonellales;Veillonellaceae;Dialister;Dialister_micraerophilus* | 0.088±0.333 | 0.286±0.232 | 0.000 |
| *Bacteria;Bacteroidetes;Bacteroidia;Bacteroidales;Prevotellaceae;Prevotella;Prevotella_melaninogenica* | 0.000±0.002 | 0.148±0.323 | 0.000 |
| *Bacteria;Saccharibacteria_TM7;Saccharimonas_c;*  *Saccharimonas_o;Saccharimonas_f;AF125206_g;DQ666092_s* | 0.000 | 1.728±4.887 | 0.000 |
| *Bacteria;Tenericutes;Mollicutes;Mycoplasmatales;*  *Mycoplasmataceae;Mycoplasma_g4;Mycoplasma_hominis* | 0.000 | 1.432±2.572 | 0.000 |
| *Bacteria;Firmicutes;Clostridia;Clostridiales;Ruminococcaceae;*  *Mageeibacillus;Mageeibacillus_indolicus* | 0.000 | 0.012±0.034 | 0.000 |
| *Bacteria;Actinobacteria;Actinobacteria_c;Actinomycetales;*  *Actinomycetaceae;Mobiluncus;Mobiluncus_mulieris* | 0.000 | 0.583±1.651 | 0.000 |
| *Bacteria;Bacteroidetes;Bacteroidia;Bacteroidales;Porphyromonadaceae;Porphyromonas;Porphyromonas_asaccharolytica* | 0.000 | 0.039±0.111 | 0.000 |
| *Bacteria;Firmicutes;Negativicutes;Veillonellales;Veillonellaceae;*  *Megasphaera;AFUG_s* | 0.002±0.022 | 0.039±0.112 | 0.043 |
| *Bacteria;Firmicutes;Erysipelotrichi;Erysipelotrichales;*  *Erysipelotrichaceae;Bulleidia;Bulleidia_extructa* | 0.000±0.002 | 0.032±0.085 | 0.000 |
| *Bacteria;Actinobacteria;Actinobacteria_c;Bifidobacteriales;*  *Bifidobacteriaceae;Bifidobacterium;Bifidobacterium_commune* | 0.000±0.002 | 0.006±0.017 | 0.043 |
| *Bacteria;Firmicutes;Bacilli;Lactobacillales;Lactobacillaceae;*  *Lactobacillus;Lactobacillus_rodentium* | 0.000 | 0.004±0.011 | 0.000 |
| *Bacteria;Tenericutes;Mollicutes;Entomoplasmatales;*  *Spiroplasmataceae;Spiroplasma; Spiroplasma_leucomae* | 0.000±0.003 | 0.001±0.004 | 0.018 |

(D)

| ***Gardnerella vaginalis*** | Negative ( n=47 ) | Positive (n=129 ) | *p*-value |
| --- | --- | --- | --- |
| *Bacteria;Firmicutes;Bacilli;Lactobacillales;Lactobacillaceae;*  *Lactobacillus;Lactobacillus_crispatus* | 53.792±49.266 | 40.956±46.701 | 0.015 |
| *Bacteria;Firmicutes;Bacilli;Lactobacillales;Lactobacillaceae;*  *Lactobacillus;Lactobacillus_iners* | 29.225±43.541 | 33.438±40.564 | 0.157 |
| *Bacteria;Actinobacteria;Actinobacteria_c;Bifidobacteriales;Bifidobacteriaceae;Gardnerella;ADEP_s* | 0.003±0.010 | 5.676±13.057 | 0.000 |
| *Bacteria;Actinobacteria;Actinobacteria_c;Bifidobacteriales;Bifidobacteriaceae;Gardnerella;ADET_s* | 0.000±0.001 | 2.478±10.931 | 0.000 |
| *Bacteria;Actinobacteria;Coriobacteriia;Coriobacteriales;*  [*Atopobiaceae*](https://en.wikipedia.org/wiki/Atopobiaceae)*;Fannyhessea;Fannyhessea vaginae* | 2.085±14.267 | 2.676±9.174 | 0.003 |
| *Bacteria;Actinobacteria;Actinobacteria_c;Bifidobacteriales;Bifidobacteriaceae;Bifidobacterium;Bifidobacterium_dentium* | 2.429±14.199 | 0.000±0.002 | 0.026 |
| *Bacteria;Actinobacteria;Coriobacteriia;Coriobacteriales;*  *Coriobacteriaceae;KQ959671_g;KQ959671_s* | 0.000 | 0.2556±0.983 | 0.006 |
| *Bacteria;Bacteroidetes;Bacteroidia;Bacteroidales;Prevotellaceae;*  *Prevotella;Prevotella_bivia* | 0.002±0.005 | 0.498±3.756 | 0.003 |
| *Bacteria;Firmicutes;Negativicutes;Veillonellales;Veillonellaceae;*  *Megasphaera;ADGP_s* | 0.000 | 0.556±1.592 | 0.001 |
| *Bacteria;Bacteroidetes;Bacteroidia;Bacteroidales;Prevotellaceae;*  *Prevotella;Prevotella_buccalis* | 0.000 | 0.535±3.432 | 0.015 |
| *Bacteria;Bacteroidetes;Bacteroidia;Bacteroidales;Prevotellaceae;*  *Prevotella;Prevotella_disiens* | 0.002±0.008 | 0.010±0.044 | 0.042 |
| *Bacteria;Bacteroidetes;Bacteroidia;Bacteroidales;Prevotellaceae;*  *Prevotella;Prevotella_timonensis* | 0.000 | 0.144±0.798 | 0.007 |
| *Bacteria;Fusobacteria;Fusobacteria_c;Fusobacteriales;*  *Leptotrichiaceae;Sneathia;Leptotrichia_amnionii* | 0.000 | 0.187±1.356 | 0.024 |
| *Bacteria;Firmicutes;Negativicutes;Veillonellales;Veillonellaceae;*  *Dialister:KQ960846_s* | 0.001±0.004 | 0.276±1.018 | 0.000 |
| *Bacteria;Firmicutes;Bacilli;Lactobacillales;Aerococcaceae;Aerococcus;*  *Aerococcus_christensenii* | 0.000 | 0.165±0.592 | 0.001 |
| *Bacteria;Firmicutes;Tissierellia;Tissierellales;Peptoniphilaceae;*  *Parvimonas;KQ959647_s* | 0.000 | 0.067±0.362 | 0.031 |
| *Bacteria;Firmicutes;Negativicutes;Veillonellales;Veillonellaceae;*  *Dialister;Dialister_micraerophilus* | 0.000 | 0.120±0.374 | 0.005 |
| *Bacteria;Firmicutes;Bacilli;Lactobacillales;Lactobacillaceae;*  *Lactobacillus;Lactobacillus_reuteri* | 0.013±0.056 | 0.068±0.168 | 0.004 |
| *Bacteria;Proteobacteria;Alphaproteobacteria;Sphingomonadales;*  *Sphingomonadaceae ; Sphingomonas;Sphingomonas_ginsenosidimutans* | 0.006±0.037 | 0.000 | 0.019 |
| *Bacteria;Firmicutes;Bacilli;Lactobacillales;Streptococcaceae;*  *Streptococcus;GL698454_s* | 0.023±0.145 | 0.000±0.000 | 0.019 |
| *Bacteria;Firmicutes;Tissierellia;Tissierellales;Peptoniphilaceae;*  *Fenollaria;LN898229_s* | 0.001±0.004 | 0.010±0.038 | 0.028 |
| *Bacteria;Firmicutes;Negativicutes;Veillonellales;Veillonellaceae;*  *Diaslister;KQ960846_s* | 0.000 | 0.002±0.010 | 0.000 |
| *Bacteria;Firmicutes;Tissierellia;Tissierellales;Peptoniphilaceae;*  *Anaerococcus;Anaerococcus_tetradius* | 0.000 | 0.015±0.082 | 0.019 |
| *Bacteria;Firmicutes;Clostridia;Clostridiales;Lachnospiraceae;*  *Howardella;AF385567_s* | 0.004±0.030 | 0.004±0.012 | 0.038 |
| *Bacteria;Firmicutes;Clostridia;Clostridiales;Ruminococcaceae;*  *KQ959578_g;AY958888_s AY958888_s* | 0.000 | 0.005±0.025 | 0.019 |
| *Bacteria;Proteobacteria;Gammaproteobacteria;Pseudomonadales;*  *Moraxellaceae;Acinetobacter;Acinetobacter_pittii* | 0.002±0.009 | 0.000 | 0.019 |
